# Case of Labour, Complicated with Rectovaginal Hernia

**Published:** 1844-10-05

**Authors:** 


### CASE OF LABOUR, COMPLICATED WITH RECTO-VAGINAL HERNIA.

By Professor Meigs.

To the Editor of the Medical Examiner.

*Sir*,—Mrs. R., aged about 30 years, the mother of four children, all of which were born by easy natural labours, and one of them in a labour of two hours, was seized with the parturient pains at half-past eleven o'clock last night. She was at full term, and in good health, save that she had complained much of an unusual pain in the right side of the abdomen, and particularly in the right iliac region.

Her physician, Dr. Bicknell, was called to the charge of the case;—Dr. B. discovered a tumour occupying the cavity of the pelvis, which impeded the progress of the labour. The woman's pains were frequent, and violent, and attended with the most excessive tenesmic effort at bearing down. Dr. B. invited me to see the patient, and I arrived at 2 o'clock, P. M., at her house in West Philadelphia.

The external parts were in a relaxed state. The index finger used in touching was pressed towards the symphisis pubis, by the tumour which seemed nearly to fill up the pelvic cavity and effectually to debar the head even from engaging in the superior strait, though the labor had continued already 14½

hours, in the case of a woman who in all other labours was occupied but two hours with the whole process.

I could just conveniently touch the presenting part of the head, which was in the 4th position of the vertex presentation. The os uteri fully dilated.

The tumour was compressible. I touched by the rectum, and so discovered that the tumour was in the peritoneal cul de sac betwixt the rectum and vagina, but distending that cul de sac enormously. The diagnosis could be nothing else, considering the softness of the swelling, than a vaginal enterocele, which I immediately proceeded to reduce.

The woman was placed on her left side. The knees drawn up. I introduced the fingers of the right hand into the passage and pressed the ends of them against the lower part of the tumour. By keeping up the pressure a short time, during which I repeatedly exhorted the woman to be passive, and not to bear down at all, I could cause the whole mass of the swelling to rise up towards the back part of the superior strait. As the mass ascended, it grew smaller, until on a sudden, the whole tumour slipped beyond the reach of the hand, and was lost. I announced this good fortune to the patient, and exhorted her not to bear down at all with the approaching pain lest the gut should again prolapse. The pain that ensued brought the head nearly through the superior strait and partially rotated the vertex. The second pain rotated the head and propelled it on the perineum; the third brought the vertex considerably beyond the pubic arch, and the fourth expelled a very large and healthy child; after which the placenta came off in a few minutes.

I look upon this as a very interesting case; not merely on account of the rareness of vaginal enterocele in the pregnant female, but as exhibiting the power of such a tumour to suspend and impede the progress of a labour in all other regards natural and healthy.

I presume, as so many hours had already elapsed in vain and exhausting efforts by a strong woman, that there was reason to fear a dangerous strangulation or contusion of the displaced bowel; and that it was fortunate for the patient that the intestine could be returned above the plane of the strait. The rapidity with which the head passed through the whole pelvis and the soft parts, as soon as the obstruction was removed, showed conclusively that the vaginal enterocele was the cause of her distress. As I have never met with such a case before, I thought that the publication of it might prove useful to some of the younger of your readers, should one of them happen to meet hereafter with a similar instance of difficulty.

I am, sir, very faithfully,
Your obedient servant,
CHARLES D. MEIGS.

*Philadelphia*, Sept. 26, 1844.

The above is an instructive narrative; one which the young practitioner ought to remember, on account of the diagnosis and treatment which it so well sets forth. A similar case occurred to us many years ago, which embarrassed us at the time exceedingly. It was not until we had traced the tumour with the finger several times, without finding any connection with the cavity of the uterus, that we became satisfied that it was not the ovum itself. We have delivered the same lady three times, and in every instance were obliged to push the protruded intestine above the fœtal head, before labour could proceed satisfactorily. In one other case of the kind which we have seen, the tumour was much smaller, and opposed no obstacle to delivery.—EDITOR.

